# Supplementary material for: Dopamine Photochemical Behaviour under UV Irradiation
Source: Int J Mol Sci. 2022 May 13;23(10):5483. doi: 10.3390/ijms23105483 (PMC9141693; doi:10.3390/ijms23105483)

Supporting Information Material

Dopamine photochemical behavior under UV irradiation

Alexandra Falamaș[1], Anca Petran[1], Alexandru-Milentie Hada[2,3] and Attila Bende[1,]*

[1]National Institute for Research and Development of Isotopic and Molecular Technologies, Donat Street, No. 67-103, Ro-400293, Cluj-Napoca, Romania.

[2]Nanobiophotonics and Laser Microspectroscopy Center, Interdisciplinary Research Institute in Bio-Nano-Sciences, Babes-Bolyai University, 42 T. Laurian Str., 400271, Cluj-Napoca, Romania

[3]Faculty of Physics, Babes-Bolyai University, 1 M. Kogalniceanu str., 400084, Cluj-Napoca, Romania

*Correspondence: attila.bende@itim-cj.ro

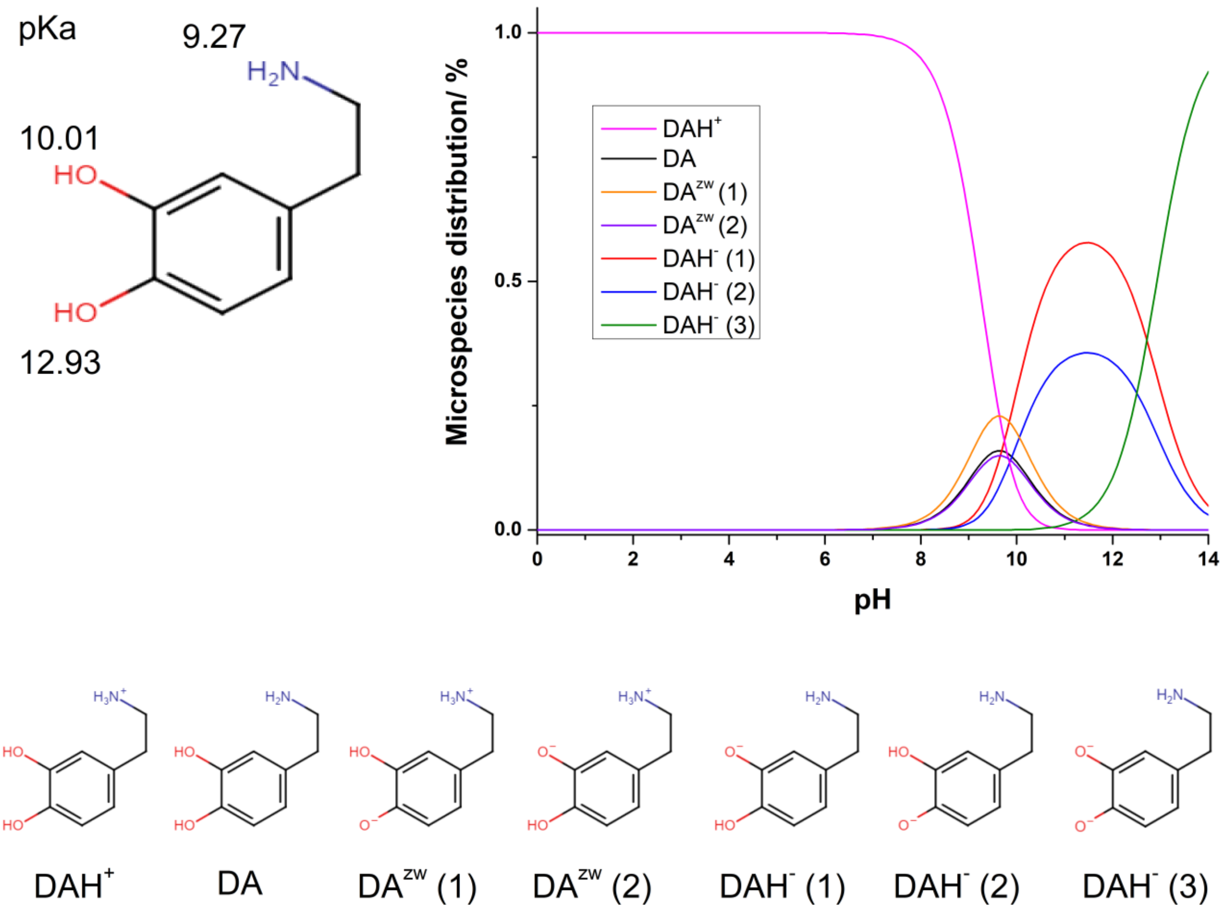

Figure S1. pH distribution of the different conformation of dopamine.

Table S1. The vibrational frequencies (ω), the Huang-Rhys factors (HRF) and the reorganization energies (λ) as well as the graphics of the vibrational amplitudes of the corresponding normal modes for the four dopamine conformations.

	DA			DA^{zw}			DAH^{+}			DAH^{-}		
	ω (cm^{-1})	HRF	λ (cm^{-1})	ω (cm^{-1})	HRF	λ (cm^{-1})	ω (cm^{-1})	HRF	λ (cm^{-1})	ω (cm^{-1})	HRF	λ (cm^{-1})
1	749	0.179	145	1225	0.107	134	753	0.202	164	1245	0.080	102
2	795	0.118	105	1437	0.112	166	798	0.109	99	1430	0.105	148
3	1355	0.198	269	1547	0.107	175	1366	0.112	152	1547	0.174	281
4	1455	0.108	151				1449	0.109	157			

DA: 749 cm^{-1}:

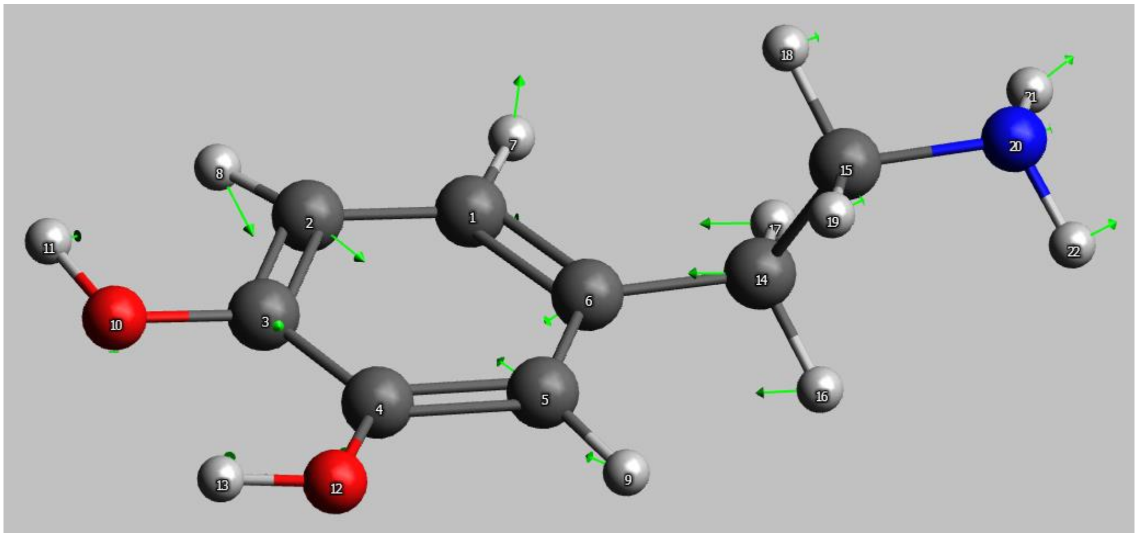

DA: 795 cm^{-1}:

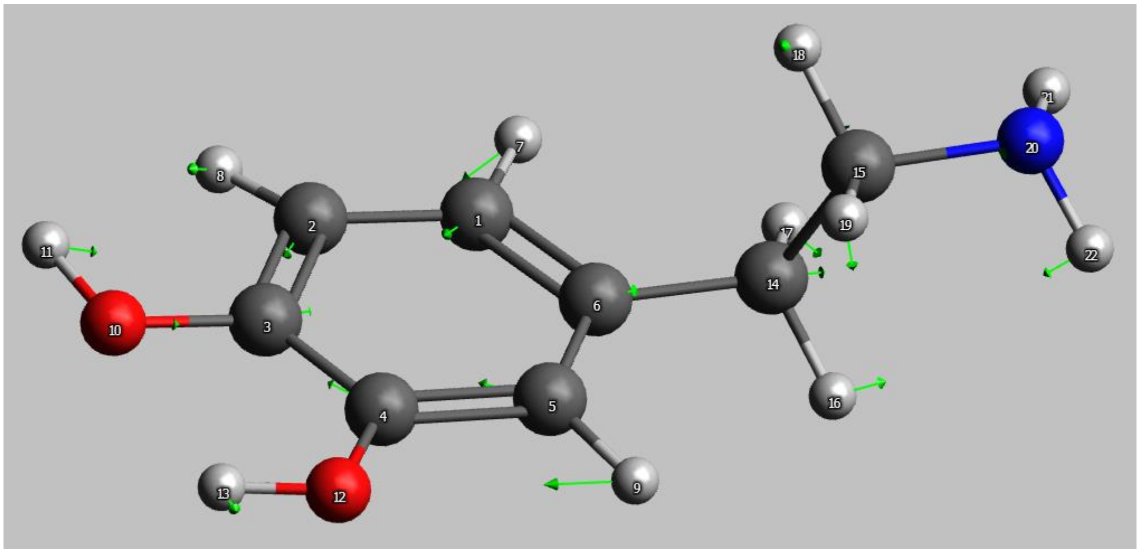

DA: 1355 cm^{-1}:

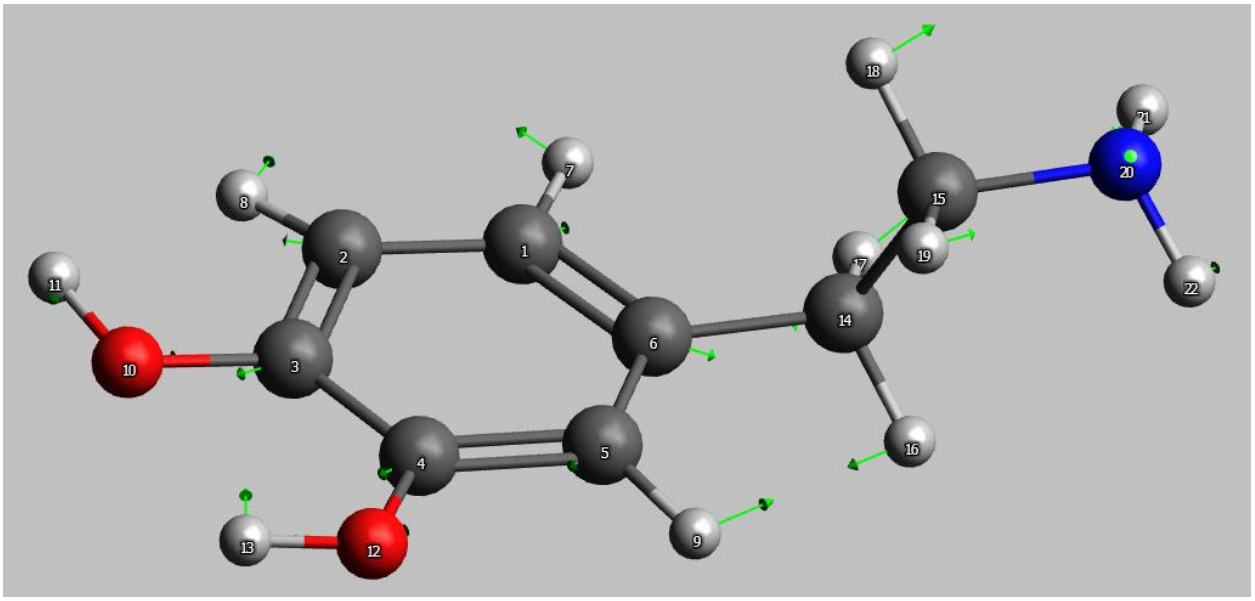

DA: 1455 cm^{-1}:

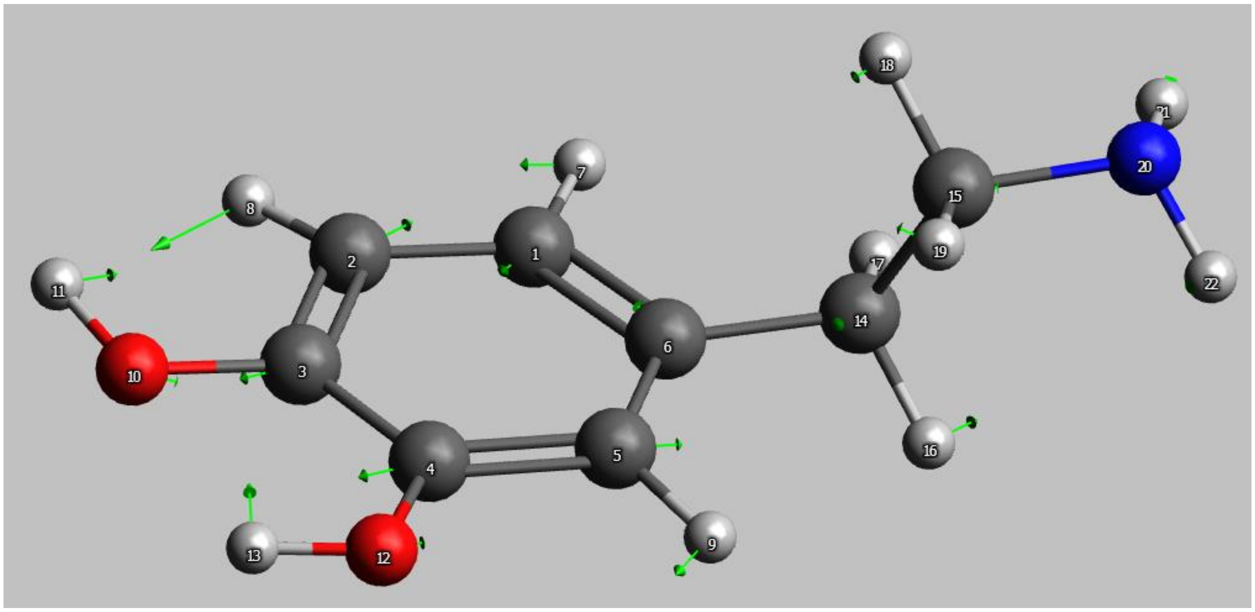

DA^{zw} 1225 cm^{-1}:

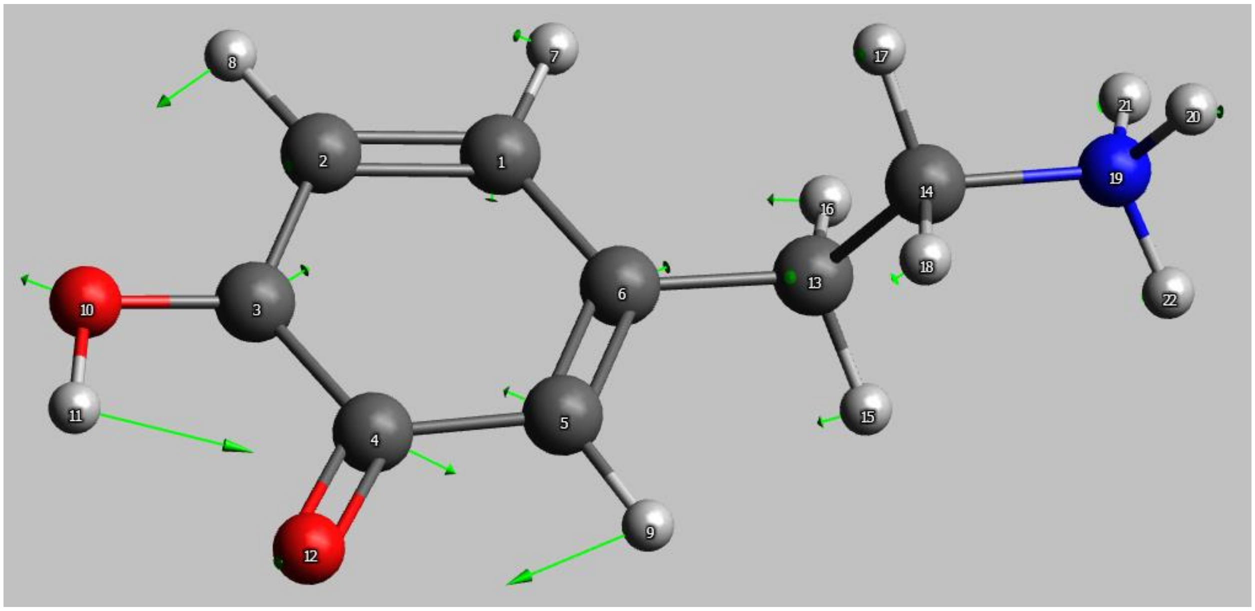

DA^{zw} 1437 cm^{-1}:

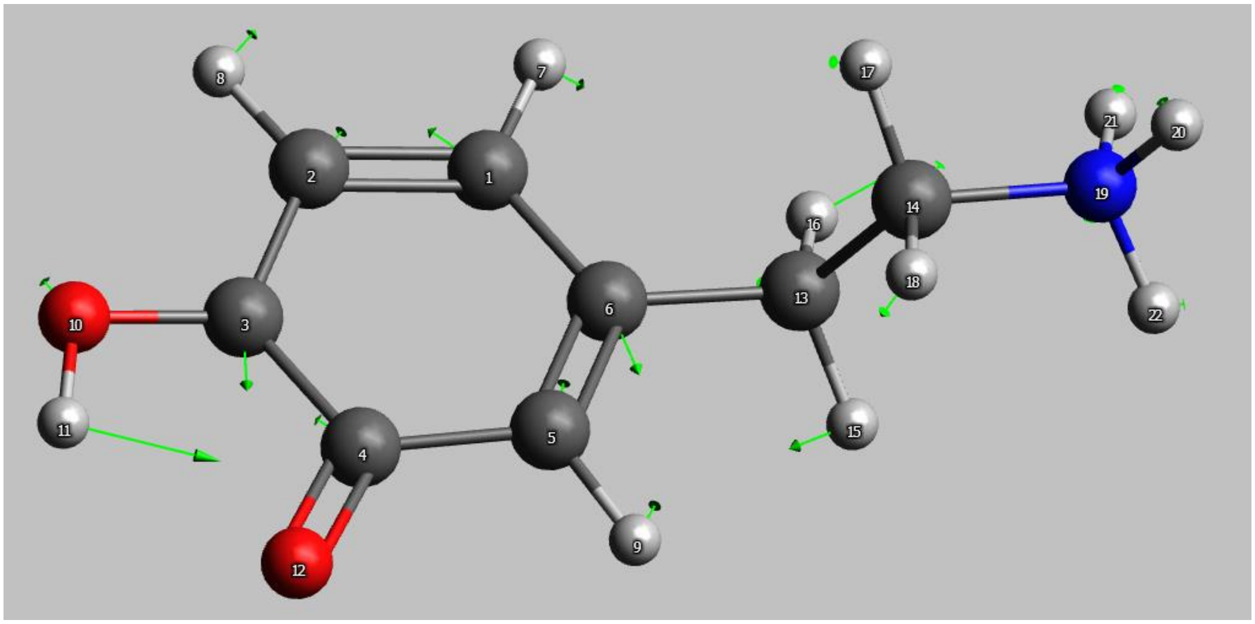

DA^{zw} 1547 cm^{-1}:

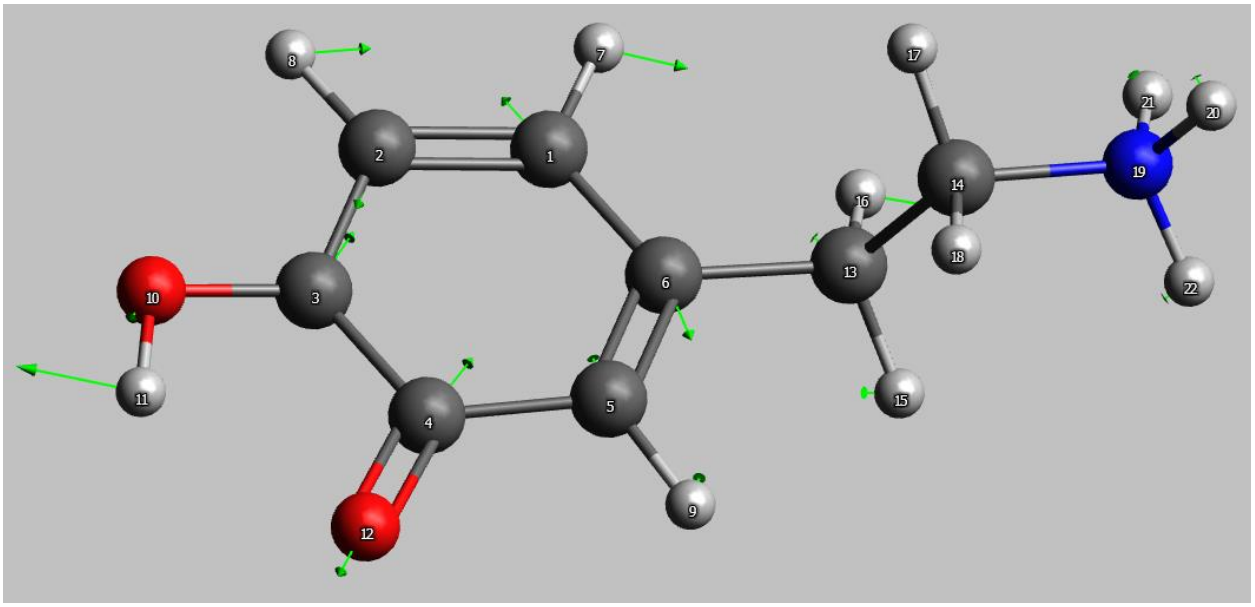

DAH^{+} 753 cm^{-1}:

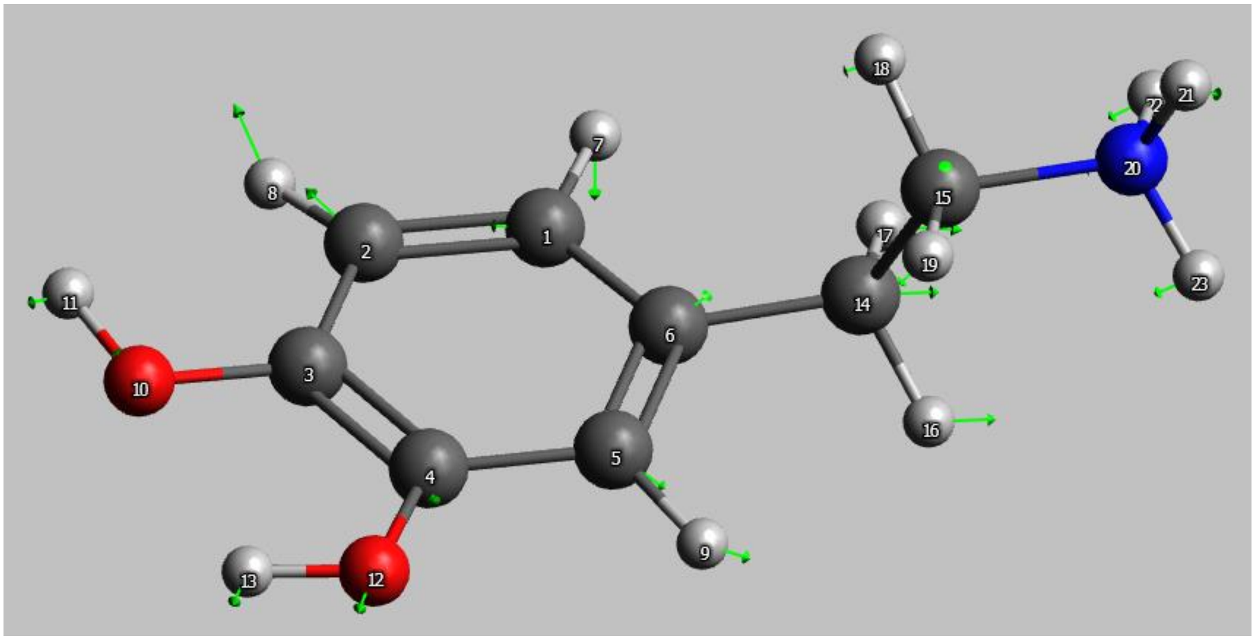

DAH^{+} 798 cm^{-1}:

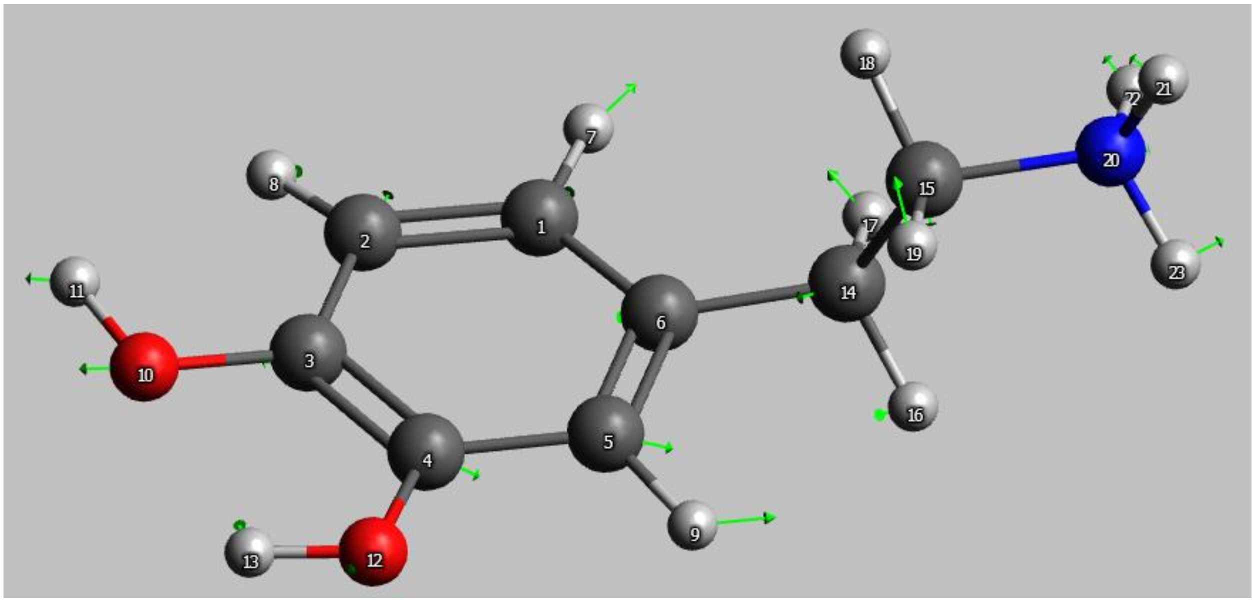

DAH^{+} 1366 cm^{-1}:

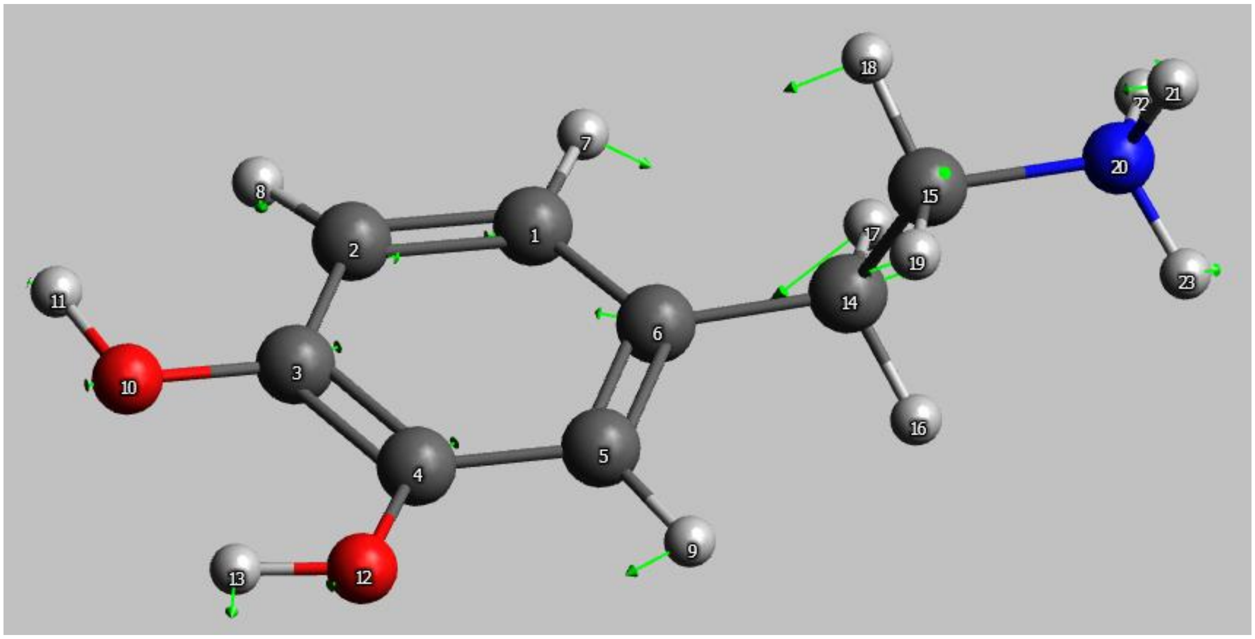

DAH^{+} 1449 cm^{-1}:

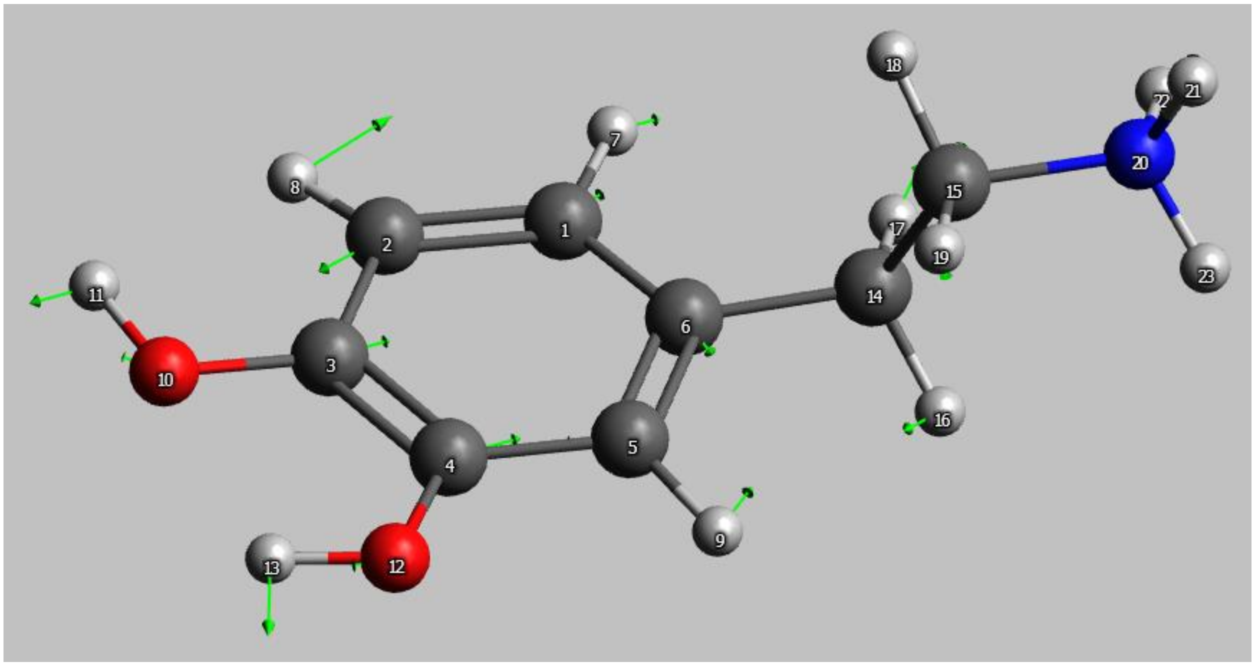

DAH^{-} 1245 cm^{-1}:

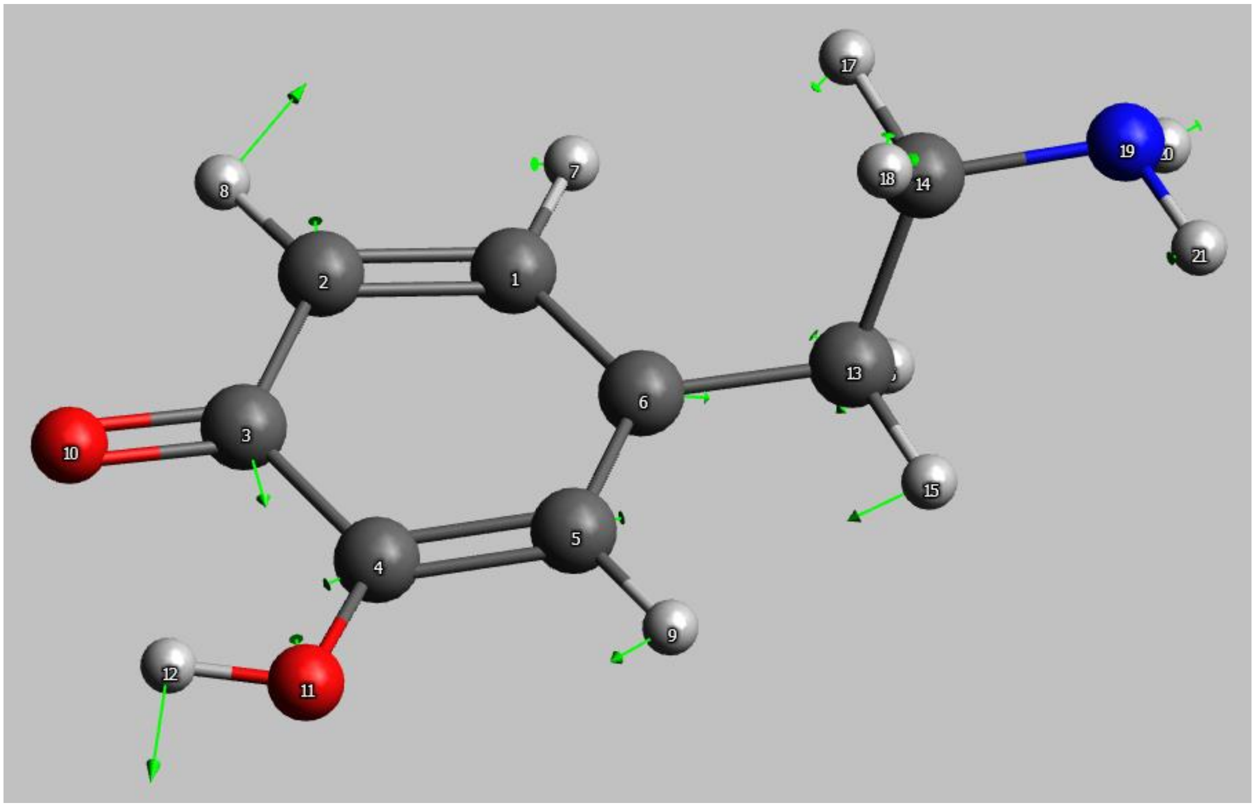

DAH⁻ 1430 cm^{-1}:

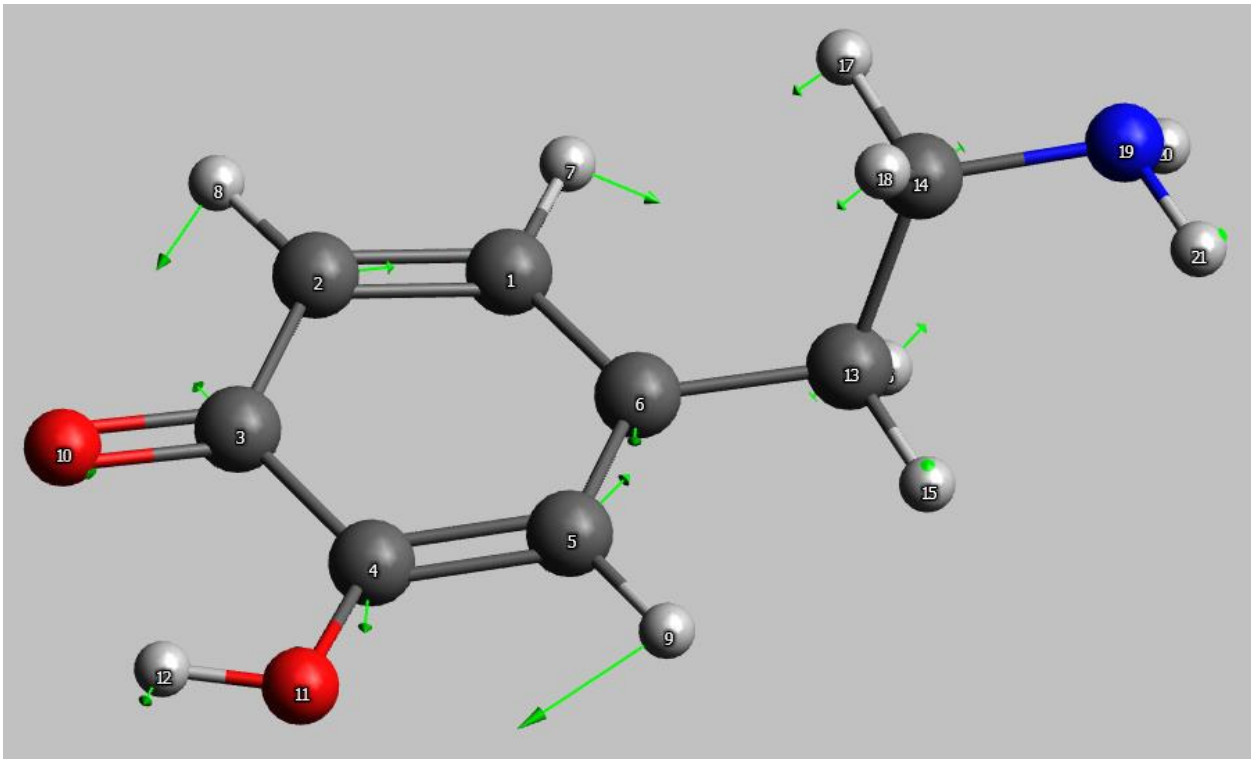

DAH⁻ 1547 cm^{-1}:

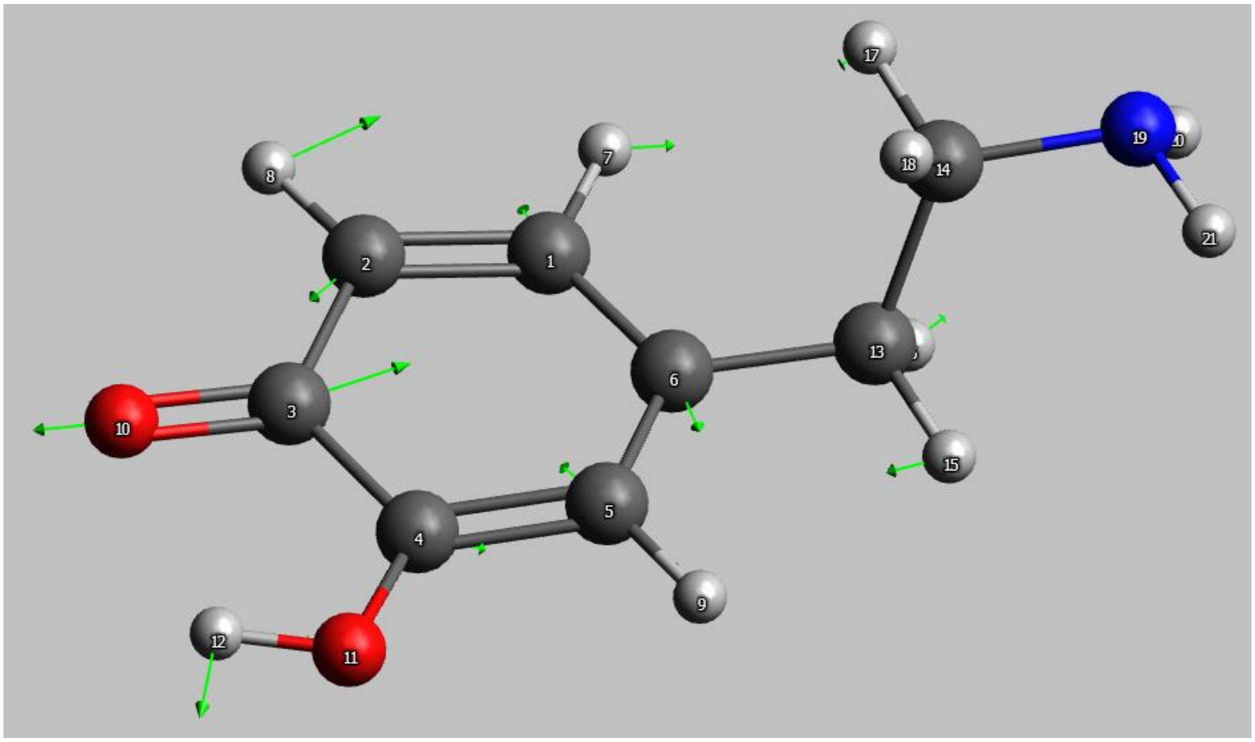

Supplement: Supplementary file 1 [file ijms-23-05483-s001.zip › ijms-1644118-supplementary.pdf]
